# Investigation of the Effects of Monosodium Glutamate on the Embryonic Development of the Eye in Chickens

**DOI:** 10.3390/vetsci10020099

**Published:** 2023-01-30

**Authors:** Ferhan Bölükbaş, Yasemin Öznurlu

**Affiliations:** 1 Department of Histology and Embryology, Faculty of Medicine, Aksaray University, 68100 Aksaray, Turkey; 2 Department of Histology and Embryology, Faculty of Veterinary Medicine, Selcuk University, 42100 Konya, Turkey

**Keywords:** monosodium glutamate, retina, cornea, eye, ganglion cells, chicken embryos

## Abstract

**Simple Summary:**

Monosodium glutamate (MSG, E62, C5H8NO4Na) is the most widely used food additive in the world for enhancing flavour. The number of flavour-enhancers added to foods is very important for the health of consumers. It has been determined by many researchers that MSG has negative effects on various organs of the body. Since chicken embryos develop without the influence of the maternal organism and allow toxicity to be evaluated very quickly and precisely, they have become a preferred experimental model for investigating the embryotoxic and teratogenic effects of chemicals, toxins, drugs, and numerous food additives and flavour-enhancing agents. This study aimed to investigate the effects of in ovo MSG administration at different doses on the embryonic development of the eye using histological and histometric techniques. The results of the present work have shown that in ovo-administered MSG can adversely affect embryonic eye development.

**Abstract:**

MSG is the most ubiquitous food additive in the food industry. The aim of this report was to investigate the effects of in ovo MSG administration on embryonic chicken eye development using histological and histometric methods. A total of 410 fertilized eggs obtained from Babcock Brown laying hens (Gallus gallus domesticus) were used and divided into 5 groups: I (untreated control), II (vehicle control), III (0.12 mg/g egg MSG), IV (0.6 mg/g egg MSG), and V (1.2 mg/g egg MSG), and injections were performed via the egg yolk. At incubation day 15, 18, and 21, 6 embryos from each group were sacrificed by decapitation and pieces of eye tissue were obtained. In all MSG groups, it was determined that both corneal epithelium thickness and total corneal thickness decreased at incubation time points 15, 18, and 21 days compared with the controls (*p* < 0.05). The total retinal thickness, thickness of the outer nuclear layer (ONL), inner nuclear layer (INL), ganglion cell layer (GL), and nerve fibre layers (NFL), as well as the number of ganglion cells decreased significantly at incubation days 15, 18, and 21 (*p* < 0.05), and degenerative changes such as vacuolar degeneration and retinal pigment epithelial detachment were also observed. In conclusion, MSG in ovo administration can affect the cornea and distinct layers of retinal cells.

## 1. Introduction

Monosodium glutamate (MSG) has been used extensively to enhance the flavour of seasonings in the food industry and restaurants. MSG can be found in processed food without an indication on the label, especially to increase the consumption and flavour of processed food such as snack food, chips, sauces, and soups [1,2]. Natural form glutamic acid in food is not toxic, but industrially produced synthetic glutamic acid is a toxin [3]. MSG is available without limits in a wide variety of processed food. It is also added to meals in unlimited quantities in restaurants, hospitals, nursing homes, and cafeterias. Food manufacturers do not list the amount of MSG on their packaging, so there is no way to know how much MSG an adult or child consumes in a day. According to industrial research, 0.6% MSG is optimally added to food [4]. According to the U.S. Environmental Protection Agency, the use of MSG in food should not be allowed for infants and especially children under one year of age [4].

Glutamate is the primary neurotransmitter stimulant for brain networks and stimulates respective receptors, which have important effects in both physiological and pathological processes [5]. Excessive stimulation of glutamate receptors leads to the development of excitotoxicity [6]. Glutamate is metabolized to non-toxic glutamine by glutamine synthetase after being taken into Müller cells by the glutamate transporter, GLAST [7]. Glutamic acid is responsible for synaptic transmission between bipolar, photoreceptor, and ganglion cells in the retina, and its presence in high amounts is responsible for neuronal cell death [8], such as retinal ganglion cells [9].

The toxic effects of MSG on the central nervous system, adipose tissue, hepatic tissue, reproductive organs, and liver and kidney functions were determined by several studies [2,10,11]. These studies revealed that MSG causes cytotoxicity [12,13] and oxidative damage in the liver, kidney, and various tissues [14,15,16,17,18] and increased the risk of certain cancers [16]. MSG may adversely affect immune system organs [13,18,19], the testes [20,21,22], ovarium [23,24,25,26,27], pancreas [28], spleen [29,30,31], liver [15,32,33], and cardiac tissue [2]. It has been reported that the incidence of certain diseases, such as obesity [34,35], diabetes, and Alzheimer’s is increased in people who consume foods with added MSG [36,37].

MSG can cause neurotoxicity, neurodegeneration [38], and neuroendocrine abnormalities [39]. There have been many studies investigating the negative effects of MSG on the nervous system [40,41,42,43,44,45].

Reports have demonstrated that MSG can be toxic to foetal development, children, and adults [4]. Maternal MSG exposure can result in neurotoxic effects and severe intrauterine growth retardation in rats [46]. In addition, MSG has been found to cause apoptosis and necrosis in the hippocampus of prepubertal rats [47]. Narayanan et al. [48] reported that MSG showed neurotoxic effects when administered to newborn animals at high concentrations. MSG has been reported to cause neurological damage by inducing oxidative stress and neurotoxicity, which is more severe in newborns during brain development than in adults [9]. In addition, Gim et al. [49] reported that MSG injection to newborn rats caused histopathological changes in the brain and degeneration of the cerebral cortex. Bölükbaş and Öznurlu [42] reported that in ovo MSG administration caused histopathological changes such as necrosis, neuronophagia, and gliosis in brain tissue.

Studies have shown that exposure to MSG causes significant degenerative effects on the eye and retinal layers [50,51,52,53,54]. Dénes et al. [55] revealed that subcutaneous injection of MSG into rats causes retinal degeneration and pycnosis of retinal cells. Additionally, in further study, it was reported that the density of ganglion cells in the retina was considerably reduced in rat models exposed to MSG, in comparison with control rats [56]. Praputpittaya and Wililak [57] reported that subcutaneous injection of MSG in different doses to newborn rats caused deficits in visual performance. Swelim [58] reported that low concentrations of subcutaneous MSG injections in neonate mice caused retinal damage.

It has been suggested that MSG administration in newborn rats leads to degeneration of retinal ganglion cells, as well as degeneration of the optic nerve [51,56]. It has also been reported to lead to degeneration of neurons in retinal layers, the arcuate nucleus, and various other brain regions, delaying the emergence of certain reflexes during neurobehavioral development and leading to temporary changes in reflex performance and motor coordination [59,60]. El-Sayyad et al. [61] found that MSG given orally to pregnant rats significantly reduced total retinal thickness, outer and inner nuclear layers, and photoreceptor layer thickness. It has also been reported that mother rats have retinal ganglion degeneration, loss of pigment epithelium, and vacuolization in the plexiform inner layer. The chick eye has been a common model to study embryonic development as well as eye diseases. The chicken eye has similar basic components to the human eye including the cornea, ciliary body, iris, lens, sclera, choroid, retina, and optic nerve [62,63]. While numerous studies have reported the neurotoxic effects of MSG on humans, experimental animals, and chicken embryos [38,41,42,45,64,65], there is insufficient information about the effects of MSG on the cornea and retina during the embryonic period. This study aimed to investigate the effects of in ovo MSG administration at different doses on the cornea and retina in chicken embryos using histological and histometric techniques.

## 2. Materials and Methods

### 2.1. Experimental Design and Preperation of Test Solution

For the experiments, 410 Babcock Brown fertilized eggs (50–55 g) obtained from Babcock Brown laying hens (Gallus gallus domesticus) were examined. The MSG doses were adjusted for 55 g egg weight. MSG (Sigma-Aldrich Chemical) was diluted with sterilized distilled water. MSG doses of 0.12 mg/g, 0.6 mg/g, and 1.2 mg/g egg were prepared and given in a volume of 100 μL. Prior to MSG injection, the eggs were disinfected for 15 min under steam obtained from mixing 21 g of potassium permanganate with 42 mL of formaldehyde/m^3^ in a closed cabin. The eggs were divided into 5 groups: Group I (untreated control, 40 eggs), which includes non-treated eggs; Group II (vehicle control, 62 eggs), which injected only distilled water; and three MSG-injected groups as Group III (0.12 mg/g egg MSG, 80 eggs), Group IV (0.6 mg/g egg MSG, 90 eggs), and Group V (1.2 mg/g egg MSG, 138 eggs). Embryonic deaths were considered in numbers of eggs per group. The eggs were injected just before starting the incubation period. In the injection groups, the injection sites on the eggs were wiped with 96% ethanol for further disinfection. All injections were performed in egg yolks at the beginning of the incubation period. By drilling a hole on the side of the egg with a special egg driller, the test solution was injected through a sterile insulin injector (26 Gx1/2”, Beybi) and then the hole was sealed with liquid paraffin. Incubations were carried out in an incubator (Imza Technical Equipment, Konya, Turkey), under optimal conditions (37.5 °C temperature and 65% relative humidity). The eggs were subjected to turning angles of 45 with a turning frequency of 12 times daily.

### 2.2. Tissue Sampling and Histologic Procedures

At incubation days 15, 18, and 21, 6 embryos from each group were sacrificed by decapitation. The stages of development of the living embryos were determined according to the Hamburger–Hamilton (1951) scale (H–H scale) [66]. Eye tissue samples were collected from embryos and fixed in 10% formalin for 24 h and then subjected to dehydration and paraffin embedding. For routine histological examination, tissue sections were stained with Crossmon’s trichrome [67], Toluidine blue, and Hematoxylin and Eosin (H&E) staining [68]. Histometric measurements were performed in serial sections taken from the eye tissue obtained on incubation days 15, 18, and 21. The thickness of the total cornea and corneal epithelium were measured from three different regions of the cornea. The thickness of total retina and retinal layers were measured from four different retina regions. Additionally, the ganglion cell number was determined by counting the number on a 100 μm line length in three different fields per section. All evaluations were performed by two researchers blinded to the sample identification.

Next, prepared sections were examined with a Leica DM-2500 model light microscope. Digital images of the required areas were captured using an attached DFC-320 model camera and analysed for histometric measurements. All histometric measurements were performed using the Leica IM50 measurement program (Leica, Leica Microsystems GmbH, Wetzlar, Germany) and numerical data of the investigated parameters were obtained.

### 2.3. Statistical Analysis

The collected data were given in mean ± SD. A *p*-value of less than 0.05 was considered statistically significant. Total corneal thickness, corneal epithelial thickness, total retinal thickness, retinal layers thickness, and the number of ganglion cells were evaluated with ANOVA and the Tukey test. This was performed by using SPSS 26 (SPSS, IBM Corp. Released 2019, Armonk, NY, USA).

## 3. Results

### 3.1. Incubation Day 15

**Figure 1:**
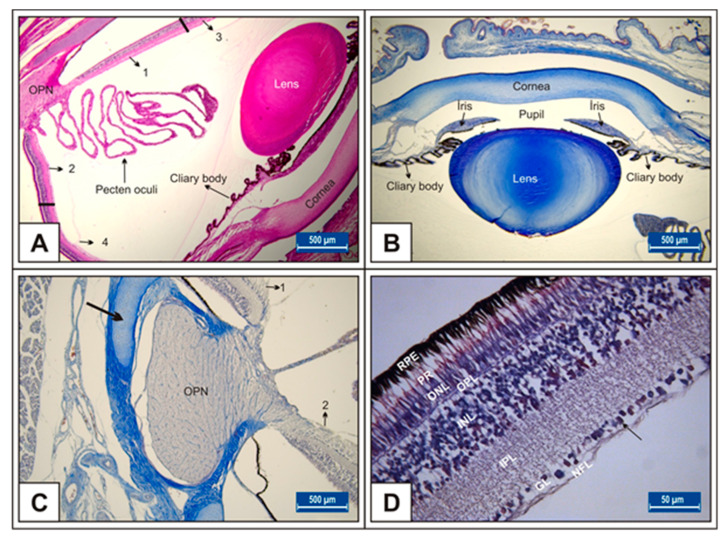
Eye histological section in Group I on 15th day of incubation (**A**); 18th day of incubation (**B**); 21st day of incubation (**C**,**D**); retinal layers (**D**); OPN: optic nerve, thick arrow: sclera with visible hyaline cartilage; arrow: Ganglion cells. Toluidine blue (**B**,**C**) and H&E (**A**,**D**) stains. Bar: 500 μm (**A**–**C**). Bar: 50 μm (**D**).

On day 15 of incubation, the transparent structures that refract and focus light (cornea and lens), muscle structures (iris and ciliary body), retina, pecten oculi, and optic nerve have developed (Figure 1A–D).

**Figure 2:**
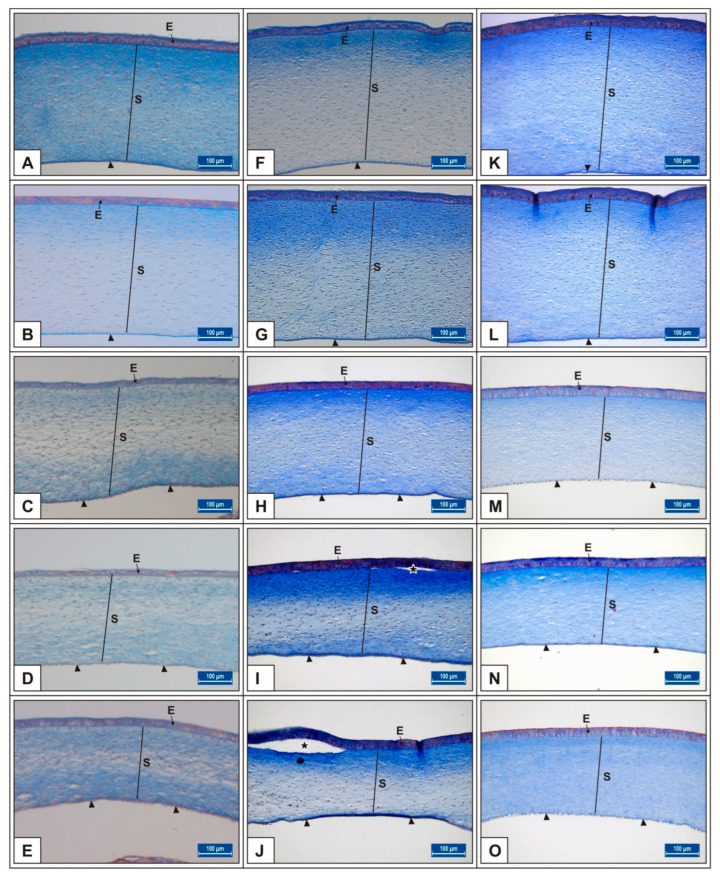
Comparison of chicken corneal layer thicknesses among Group I, II, III, IV, and V on the 15th (**A**–**E**), 18th (**F**–**J**), and 21st (**K**–**O**) days of incubation. Groups I (**A**,**F**,**K**), II (**B**,**G**,**L**), III (**C**,**H**,**M**), IV (**D**,**I**,**N**), and V (**E**,**J**,**O**) on days 15, 18, and 21 of incubation, respectively. Arrowhead: endothelium; E→: epithelium, S: stroma; stars (*): detachment of the epithelium layers. Crossmon’s triple staining. Bar: 100 μm.

It was noted that in the MSG-treated groups, corneal epithelium thickness and total corneal thickness decreased depending on the dose in comparison to untreated and vehicle controls (*p* < 0.05, Figure 2A–E, Table 1).

**Figure 3:**
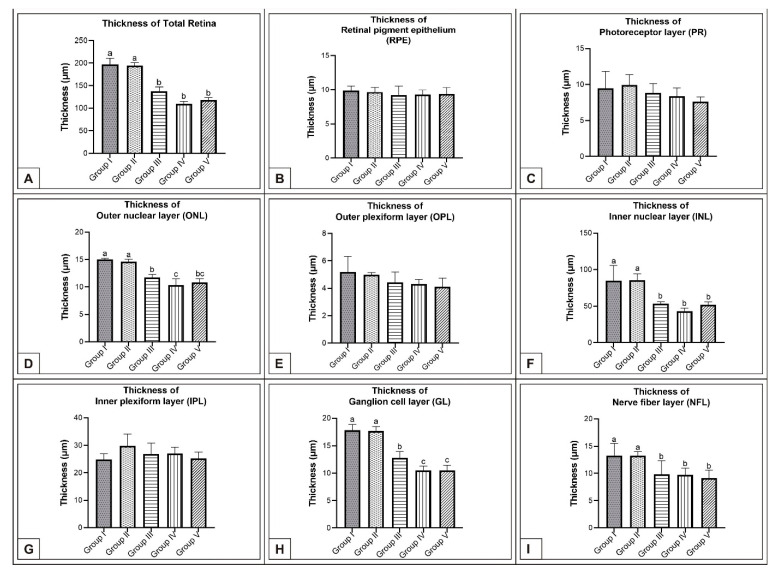
Comparison of chicken retina layer thicknesses (μm) among Group I, Group II, Group III, Group IV, and Group V on the 15th day of incubation in chicken embryos. Retinal thickness (**A**); thickness of RPE (**B**); thickness of PR (**C**); thickness of ONL (**D**); thickness of OPL (**E**); thickness of INL (**F**); thickness of IPL (**G**); thickness of GL (**H**); thickness of NFL (**I**). ^(a–c)^ Various superscript letters on the columns indicate a statistical difference (mean ± SD, *p* < 0.05).

**Figure 4:**
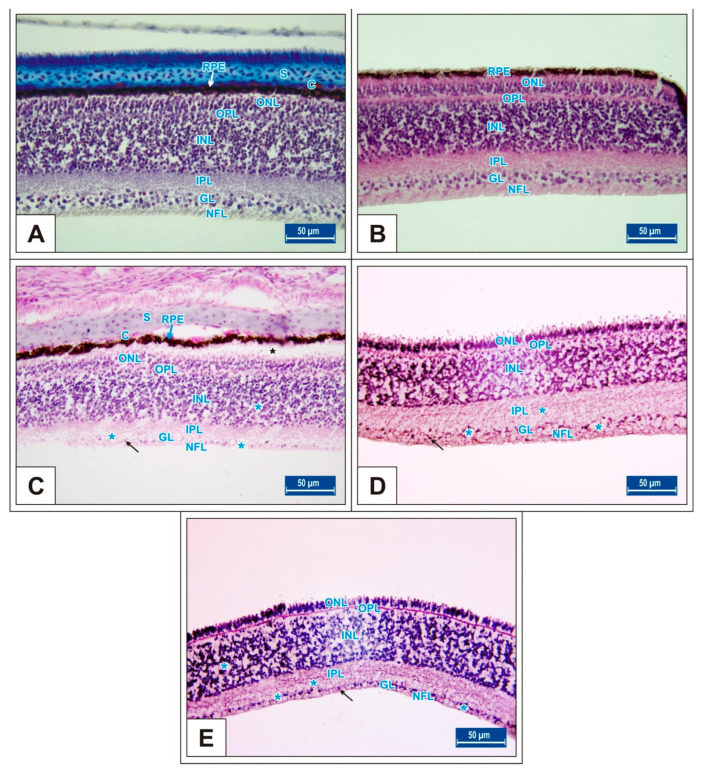
Sections from the retina of the Groups I (**A**), II (**B**), III (**C**), IV (**D**), and V (**E**) at day 15 of incubation. Blue stars (*): vacuole formation in the IPL, INL, GL, and NFL layers; black stars (*): retinal pigment epithelium detachment; arrow: ganglion cells; S: scleral cartilage; C: choroid; retinal pigment epithelium (RPE); nerve fibre layer (NFL); ganglion cell layer (GL); inner plexiform layer (IPL); inner nuclear layer (INL); outer plexiform layer (OPL); outer nuclear layer (ONL). H&E staining.

On the 15th day of incubation, it was observed that the retina consisted of 9 layers, respectively, from the outside to the inside: retinal pigment epithelium (RPE), photoreceptor layer (PR), outer limiting membrane, outer nuclear layer (ONL), outer plexiform layer (OPL), inner nuclear layer (INL), inner plexiform layer (IPL), ganglion cell layer (GL), and nerve fibre layer (NFL) (Figure 1D). In the MSG groups, total retinal thickness decreased significantly compared with the controls. Corneal epithelium thickness and total corneal thickness decreased in the MSG groups depending on the dose compared with the controls, and the thickness of the retinal layers, especially the ONL, INL, GL, and NFL layers decreased significantly (*p* < 0.05, Figure 3A–I). Furthermore, in these groups retinal degeneration such as retinal pigment epithelium detachment (Figure 4C–E) and vacuolization in the IPL, INL, GL, and NFL layers were observed (Figure 4C–E). Moreover, ganglion cell count was significantly reduced in the GL layer and this layer was thinner for all MSG groups (*p* < 0.05, Figure 4A–D, Table 2).

### 3.2. Incubation Day 18

**Figure 5:**
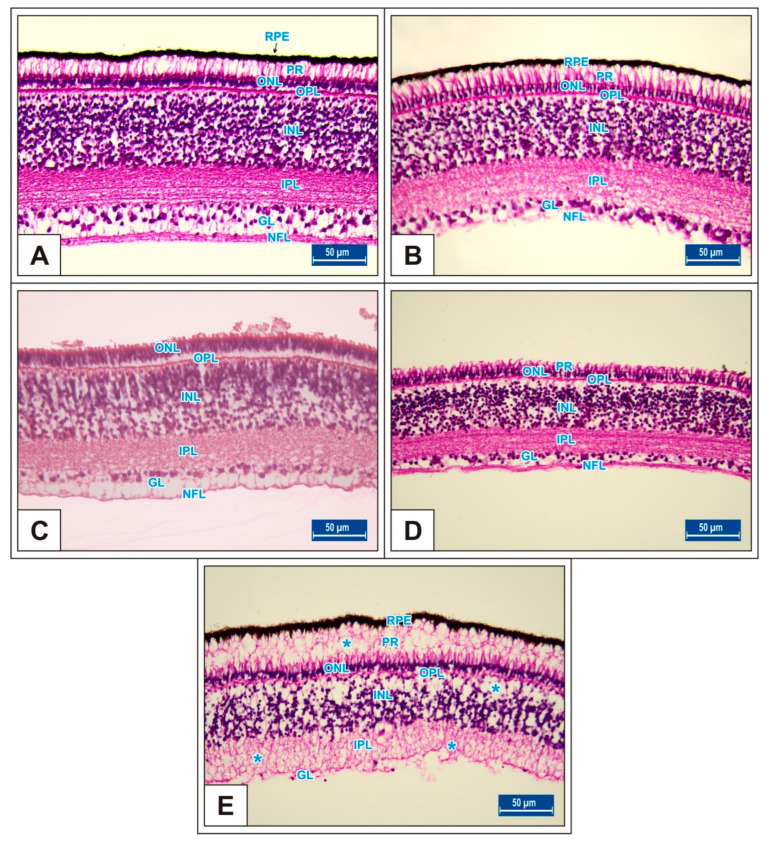
Sections from the retina of Groups I (**A**), II (**B**), III (**C**), IV (**D**), and V (**E**) at incubation day 18. Blue stars (*): vacuole formation in the PR, INL, IPL, and GL layer. Retinal pigment epithelium (RPE); photoreceptor layer (PR); nerve fibre layer (NFL); ganglion cell layer (GL); inner plexiform layer (IPL); inner nuclear layer (INL); outer plexiform layer (OPL); outer nuclear layer (ONL). H&E staining.

On incubation day 18, in MSG-treated groups retinal degenerations such as detachment of the retinal pigment epithelium (Figure 5C,D) and vacuolization in the IPL, INL, GL, and PR layer were observed (Figure 5E). Moreover, ganglion cell numbers were significantly decreased in all MSG groups compared with controls (*p* < 0.05, Table 2, Figure 5C–E).

**Figure 6:**
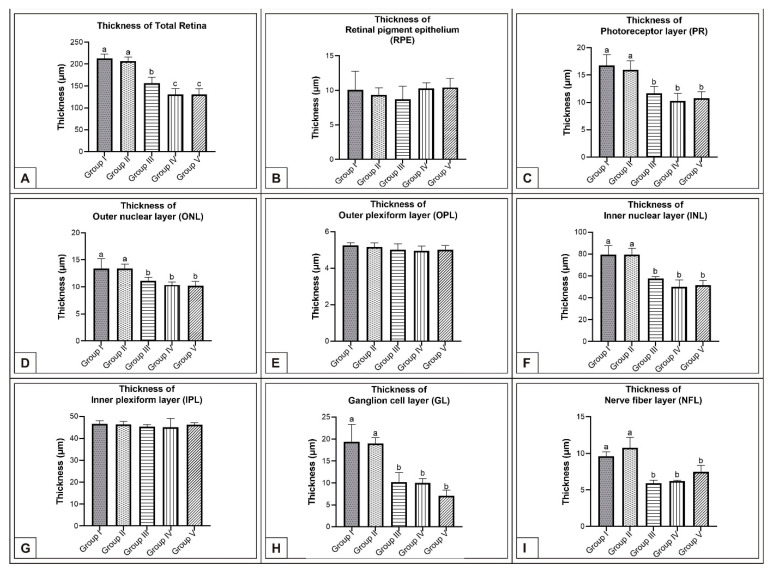
Comparison of retinal layer thicknesses (μm) among Groups I, II, III, IV, and V on incubation day 18 in chicken embryos. Retinal thickness (**A**); thickness of RPE (**B**); thickness of PR (**C**); thickness of ONL (**D**); thickness of OPL (**E**); thickness of INL (**F**); thickness of IPL (**G**); thickness of GL (**H**); and thickness of NFL (**I**). ^(a–c)^ Various superscript letters on the columns indicate a statistical difference (mean ± SD, *p* < 0.05).

In the MSG groups, corneal epithelium and total corneal thickness decreased depending on the dose in comparison with controls (*p* < 0.05, Figure 2F–J, Table 1). Similarly, the total retinal thickness decreased significantly when compared with controls; in particular, thickness of the PR, ONL, INL, GL, and NFL layers was reduced (*p* < 0.05, Figure 6A–I).

### 3.3. Incubation Day 21

**Figure 7:**
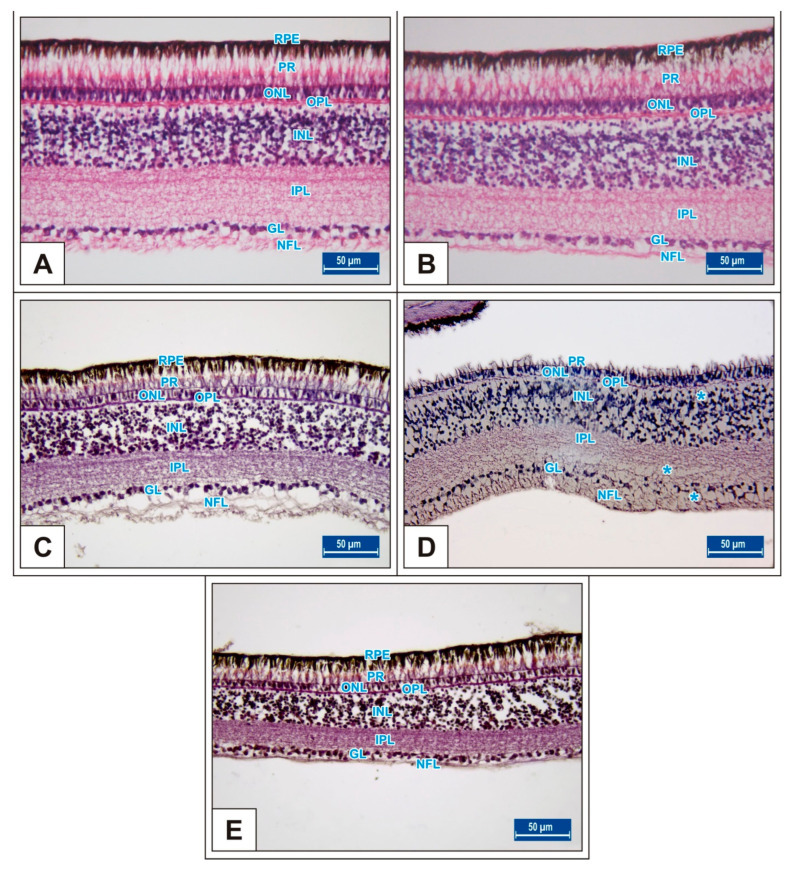
Sections from the retina of Groups I (**A**), II (**B**), III (**C**), IV (**D**), and V (**E**) at incubation day 21. Blue stars (*): vacuole formation in the INL, IPL, and NFL layers. Retinal pigment epithelium (RPE); photoreceptor layer (PR); nerve fibre layer (NFL); ganglion cell layer (GL); inner plexiform layer (IPL); inner nuclear layer (INL); outer plexiform layer (OPL); outer nuclear layer (ONL). H&E staining.

**Figure 8:**
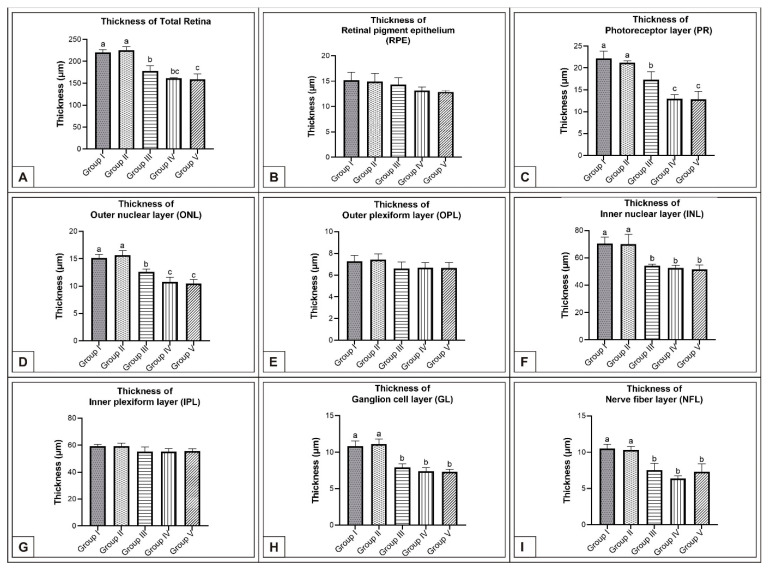
Comparison of retina layer thicknesses (μm) among Groups I, II, III, IV, and V on incubation day 21 in chicken embryos. Retinal thickness (**A**); thickness of RPE (**B**); thickness of PR (**C**); thickness of ONL (**D**); thickness of OPL (**E**); thickness of INL (**F**); thickness of IPL (**G**); thickness of GL (**H**); thickness of NFL (**I**). ^(a–c)^ Various superscript letters on the columns indicate a statistical difference (mean ± SD, *p* < 0.05).

On incubation day 21, it was determined that corneal epithelium and total corneal thickness significantly decreased following MSG treatment in comparison with controls (*p* < 0.05, Figure 2K–O, Table 1). It was also noted that total RT as well as PR, ONL, INL, GL, and NFL thickness significantly decreased when compared with controls (*p* < 0.05, Figure 7A–E, Figure 8A–I). These groups were observed to have retinal degenerations such as detachment of the RPE and cellular vacuolization of INL and GL layers (Figure 7D). Additionally, the number of ganglion cells in the GL layer of the MSG groups significantly decreased in comparison with the control groups (*p* < 0.05, Table 2).

## 4. Discussion

In this study, we studied the effects of in ovo MSG administered at different doses on the cornea and retina of chicken embryos. While a significant number of studies have reported on the various adverse effects of MSG on humans, experimental animals, and chicken embryos [38,41,42,45,64,65,69,70], no detailed studies have been found in the literature on the effects of MSG on embryonic eye development in chick embryos.

Regulations have been imposed on the use of MSG as a flavour-enhancing food additive. In August 2017, the European Food Safety Authority (EFSA) corrected the acceptable daily intake (ADI) for glutamic acid and salts from 120 mg to 30 mg per kilogram of body weight. While the average daily intake of MSG ranges from 0.3 to 0.5 g per day among European Union member countries, the average daily intake of MSG ranges are higher in Asian countries, ranging from 1.6–2.3 g/day in South Korea, 1.5–3.0 g/day in Taiwan, 1.1–1.6 g/day in Japan, and 4 g/day in China [1,3]. In recent years, large-scale shifts in lifestyle and eating habits across the globe have led to increased consumption of processed food, elevating the risk of exceeding the ADI of MSG and similar chemicals. Many reports have demonstrated the adverse effects of low dose MSG resulting from chronic consumption [1,2,71,72]. MSG doses between 1.25 and 12 gr were used in human experimental studies [73,74], dosages ranging from 0.04 to 100 g in animal studies [2,12,75,76], and dosages between 0.75 and 3 mg/g egg in chicken embryo studies [41,70]. In this study, both human daily MSG intake and previous chicken embryo studies were taken into account in the adjustment of MSG doses (0.12 mg/g, 0.6 mg/g, and 1.2 mg/g eggs).

Plasma glutamate concentration has been reported to increase significantly after MSG supplementation in humans [77]. High oral intake of MSG in rats has been shown to result in oxidative stress in multiple areas of the brain and in the retina [78,79]. This excitotoxicity effect of glutamate was reported to trigger extreme glutamate receptor (GluR) activity [80]. Ganglion cell and retina IPL have been reported to be mostly affected by both in vivo and in vitro MSG administration [55]. Van Rijn et al. [56] showed that the administration of MSG to rats significantly reduced the density of ganglion cells in the retina in comparison with controls. El-Gohari et al. [81] showed that different doses of MSG injection in rats caused retinal degeneration as well as a decrease in retinal thickness and ganglion cell count. Researchers have also reported that MSG causes detachment in the RPE and the irregular presence of the photoreceptor layer in rats. Ohguro et al. [79] reported that retinal layer thickness was thinner and the number of cells in GL, INL, and ONL decreased in rats fed three different doses of MSG. Bellhorn et al. [50] showed that MSG given to newborn rats on days 1 to 10 after birth caused degeneration of the inner retinal layers. In addition, oral administration of MSG has been reported to cause degeneration in rabbit retina [82]. AlThanoon and Abd [83] reported that different doses of MSG injection to pregnant mice caused degeneration and necrosis of the INL and ganglion cells as well as of the optic nerve depending on the dose in mouse embryos on the 14th and 18th days of pregnancy.

Chicken embryos are a common model for investigation of embryotoxicity [84,85], neurotoxicity [42,86,87], eye diseases [88], retinal development [89], and retinal pathologies [90]. Therefore, chicken embryos are good models for investigation of the effects of many food additives, flavour-enhancing additives, and environmental pollutants during embryogenesis because embryonic development is fully described and the individual developmental stages are clearly visible and easily accessible [91,92]. The chicken egg is a closed system that has no interaction with its environment, except for the interchange of gases. Jessl et al. [92] suggested that a single injection of different test solutions into the yolk sac on day one of incubation results in chronic chemical exposure and may be sufficient to influence the developing embryo. In this study, different doses of MSG (0.12 mg/g, 0.6 mg/g, and 1.2 mg/g eggs) were administered to egg yolks as a single injection, thus creating chronic exposure.

Changes in corneal thickness with age were still unclear in humans and animals. While some studies reported no significant change in corneal thickness over time [93,94], other studies showed a decreasing trend of corneal thickness with age [95,96]. Inomata et al. [97] reported that corneal thickness initially increased between 1 and 6 months, reached a maximum at 9 months, and then decreased between 12 and 14 months, while body size (weight) increased with age. AlThanoon and Abd [83] reported that, in the eyes of 18-day-old MSG-treated albino mice foetuses, there was deformation of the lens, thickening of the cornea’s inner lining, necrosis of the cornea’s stroma, and necrosis of the optic nerve. Al-Qudsi and Azzouz [98] showed that electromagnetic mobile radiation caused changes in neural retinal thickness and congenital malformations in chick embryos at 7, 10, and 14 days of incubation. In previous chick embryo retina culture studies, MSG was suggested to contribute to inner retinal layer and ganglion cell damage [99,100]. Kujawa-Hadryś et al. [101] reported that the thickness of the corneal epithelium increased markedly through the end of incubation in chick embryos. Several studies have been conducted examining normal retinal structure in poultry [102,103]. However, there is insufficient information about the effects of MSG on the cornea and retina in chicken embryos.

In this study, it was observed that corneal epithelium and total corneal thickness increased considerably from day 15 to day 21 of incubation. In the MSG groups, both corneal epithelium and total corneal thickness significantly decreased depending on the dose on days 15, 18, and 21 of incubation compared with controls, (*p* < 0.05, Figure 2, Table 1). In addition, detachment in the corneal epithelium was noted in the MSG groups. It was further noted that in the MSG groups, total retinal thickness decreased significantly on the incubation days examined compared with control groups, and thickness of both the ONL, INL, GL, and NFL layers of the retinal layers on the 15th day, and the PR, ONL, INL, GL, and NFL layers on days 18 and 21, were significantly reduced (*p* < 0.05, Figure 3A–I, Figure 6A–I and Figure 8A–I). In these groups, degenerative changes such as vacuolar degeneration and retinal pigment epithelial detachment were observed. It was also observed that the number of ganglion cells decreased markedly in all MSG groups in comparison with the control groups on all days tested (*p* < 0.05, Table 2). These findings were consistent with rat, mouse, and chicken studies [79,81,82,83,97,99,100].

This study has certain limitations that should be noted. First, it is difficult to calculate the rate of MSG uptake from the yolk sac of the developing embryo; however, it can be argued that this rate is proportional to the rate of embryo development. Moreover, the chicken embryo model has the advantage of allowing potentially hazardous chemicals to be investigated directly on the embryo. On the other hand, the detachments seen in the cornea and retina bring to mind artificial separations in the sections. However, the absence of such detachment in the control groups was evaluated as the effect of MSG.

## 5. Conclusions

It was observed that different doses of in ovo MSG administration caused histometric and histopathological changes in both the cornea and retina. Since the data obtained from studies using chicken embryos can also be adapted to mammals, the findings of this study suggest that animals and humans exposed to MSG during the prenatal period may have increased susceptibility to certain eye diseases during their lifetime. Given these results, our study adds a new perspective to the literature. Nowadays, while exposure to and/or consumption of MSG in various ways is rapidly and unsafely increasing, it may be advisable to minimize or even prohibit eating foods containing MSG, especially during pregnancy.

## Figures and Tables

**Table 1:** Corneal epithelium thickness (µm) and total corneal thickness (µm) on days 15, 18, and 21 of incubation.

Groups *n* = 6	Day 15 of Incubation		Day 18 of Incubation		Day 21 of Incubation	
	Thickness of epithelium	Total corneal thickness	Thickness of epithelium	Total corneal thickness	Thickness of epithelium	Total corneal thickness
Group I	25.22 ± 2.02 ^a^	328.42 ± 30.41 ^a^	29.68 ± 2.54 ^a^	416,70 ± 5.62 ^a^	33.12 ± 3.35 ^a^	417.85 ± 12.98 ^a^
Group II	24.99 ± 2.18 ^a^	318.59 ± 5.59 ^a^	29.24 ± 1.19 ^a^	413.51 ± 10.18 ^a^	33.33 ± 6.11 ^a^	413.05 ± 13.44 ^a^
Group III	15.96 ± 1.67 ^b^	249.22 ± 14.60 ^b^	24.72 ± 1.80 ^b^	308.73 ± 14.68 ^b^	27.16 ± 1.77 ^b^	377.58 ± 21.98 ^b^
Group IV	13.15 ± 1.71 ^c^	222.85 ± 21.31 ^c^	22.82 ± 1.15 ^c^	295.67 ± 7.16 ^c^	26.30 ± 1.45 ^b^	365.91 ± 13.43 ^bc^
Group V	13.71 ± 1.61 ^c^	210.04 ± 17.15 ^c^	22.22 ± 1.86 ^c^	291.36 ± 6.39 ^c^	24.92 ± 2.34 ^b^	359.04 ± 11.60 ^c^

^(a–c)^ Various superscript letters on the columns indicate a statistical difference (mean ± SD, *p* < 0.05).

**Table 2:** Ganglion cell numbers of the granular layer on days 15, 18, and 21 of incubation.

	Ganglion Cell Numbers		
Groups *n* = 6	Day 15 of incubation	Day 18 of incubation	Day 21 of incubation
Group I	22.58 ± 2.91 ^a^	21.47 ± 3.21 ^a^	18.94 ± 4.86 ^a^
Group II	21.01 ± 3.28 ^a^	22,52 ± 4.32 ^a^	17.58 ± 3.07 ^a^
Group III	9.71 ± 3.56 ^b^	12.74 ± 2.93 ^b^	10.84 ± 4.36 ^b^
Group IV	7.66 ± 3.04 ^b^	9.11 ± 2.76 ^c^	10.33 ± 1.93 ^b^
Group V	7.44 ± 1.56 ^b^	8.86 ± 2.94 ^c^	8.27 ± 2.78 ^b^

^(a–c)^ Various superscript letters on the columns indicate a statistical difference (mean ± SD, *p* < 0.05). Ganglion cell numbers were determined by counting the number on a 100 μm line length in three different fields per section.

## Data Availability

Not applicable.
